# The Matrix in Amalgam Fillings

**Published:** 1885-01

**Authors:** G. C. Caboll

**Affiliations:** Buffalo, N. Y.


ARTICAL III.

### THE MATRIX IN AMALGAM FILLINGS.

BY G. C. CABOLL, M.D.S., BUFFALO, N. Y.

[Read before the Seventh and Eighth District Dental Societies of New York, at Rochester, October 28, 1884.]

It should be an axiom wiht every member of the dental profession that "anything that is worth doing at all, is worth doing well." In the years past during what might be termed the rise and progress of dentristry, from empiricism to its present elevation, on which it rests a demonstrated science, and is accorded that rank, its members have devoted themselves, and justly too, to the solution of the more difficult problems that were successively presented, and ignored in many instances the claim to their attention of the simpler ones. Thus it is to-day that the art of gold filling has, by the study of the character of gold, the invention of instruments, the devising of the thousond and one accessories, reached a high degree of perfection ; but with many operators who can boast of success with gold it has been obtained to the exclusion of a proper knowledge of the working of other materials, and thus it is that many, and I might say the majority, of successful dentists do not make creditable operations with the plastic fillings. They are considered too simple ; can be done too easily. Most dentists think it does not pay to adjust the rubber dam for an operation that is going to require but a few moments to complete.

And this brings me to the subject of this short paper : "The Matrix in Amalgam Fillings." My advocacy of the use of the matrix is well known, but it has been chiefly with gold, claiming that with it approximate fillings can be introduced in less time, with greater ease, and far more certainty, than without it. I make the same claim for plastic metallic filling. It is claimed by the best authorities that the minimum of mercury in amalgam fillings insures the better result, so that with the mixture of the desired proportions the amalgam must be moulded to insure facility in handling it and in approximate cavities the introduction in this form is rendered very difficult on account of the mass resolving itself into a powder, and the the impossibility of applying sufficient force to condense it without escaping from the proximal margins.

We know that crown cavities are easier to fill than those on the proximate surfaces, easier to keep dry, easier of access, and easier to finish, and this holds just as good with plastic materials as with gold.

The operator who aims at uniformity of excellence in all his work, will not fail to avail himself of every means to secure the desired end, and to that end the matrix is an an indispensable adjunct in filling teeth with amalgam. It is not desirable to use material for its cheapness and the rapidity with which it can be worked to the exclusion of all other qualities, but rather we should aim to procure that which will bear the highest test for color, edge-strength, and freedom from shrinkage; but when we have the material that possesses all these qualities, we may fail because we neglect to use the means necessary to bring out these qualities, thus, if obliged to add an excess of mercury to render the mass sufficiently plastic to insure its introduction into a proximate cavity, we do it at the expense of strength and limit of shrinkage. If we use an amalgam that will possess sufficient strength under such conditions, we must sacrifice color. In my own practice I use Dawson's amalgam, or white alloy, as it is called, add the mercury by weight, triturate the mass in a mortar, and mould it for use, introducing it as soon as possible after preparation, frequently using the mallet in condensing and perfecting the amalgamation. After the cavity is prepared I invariably adjust the rubber dam, using the same care in the preliminaries as I would with gold, and expect to have a filling that will admit of finishing at the same sitting, one that will not shrink or discolor, and that will take a durable polished surface and stand the action of mastication.

Such a result I could not accomplish in proximate cavities without the aid of the matrix. Such a result cannot be reached without scrupulous care in all the details of the operation. With care in the selection of the be material, and the observation of all the requirements, such a thing as an artistic amalgam filling may be produced, and, although it is only an amalgam filling, "if it is worth doing at all it is worth doing well."—*The Den. Adver*

